# Digital dissection and three-dimensional interactive models of limb musculature in the Australian estuarine crocodile (*Crocodylus porosus*)

**DOI:** 10.1371/journal.pone.0175079

**Published:** 2017-04-06

**Authors:** Ada J. Klinkhamer, D. Ray Wilhite, Matt A. White, Stephen Wroe

**Affiliations:** 1 Function, Evolution and Anatomy Research Lab, School of Environmental and Rural Science, University of New England, Armidale NSW Australia; 2 Australian Age of Dinosaurs Museum of Natural History, Winton QLD Australia; 3 Department of Anatomy, Physiology and Pharmacology, College of Veterinary Medicine, Auburn University, Alabama AL United States of America; 4 School of Engineering, University of Newcastle, Callaghan NSW Australia; College of the Holy Cross, UNITED STATES

## Abstract

Digital dissection is a relatively new technique that has enabled scientists to gain a better understanding of vertebrate anatomy. It can be used to rapidly disseminate detailed, three-dimensional information in an easily accessible manner that reduces the need for destructive, traditional dissections. Here we present the results of a digital dissection on the appendicular musculature of the Australian estuarine crocodile (*Crocodylus porosus*). A better understanding of this until now poorly known system in *C*. *porosus* is important, not only because it will expand research into crocodilian locomotion, but because of its potential to inform muscle reconstructions in dinosaur taxa. Muscles of the forelimb and hindlimb are described and three-dimensional interactive models are included based on CT and MRI scans as well as fresh-tissue dissections. Differences in the arrangement of musculature between *C*. *porosus* and other groups within the Crocodylia were found. In the forelimb, differences are restricted to a single tendon of origin for *triceps longus medialis*. For the hindlimb, a reduction in the number of heads of *ambiens* was noted as well as changes to the location of origin and insertion for *iliofibularis* and *gastrocnemius externus*.

## Introduction

Investigation into the evolution and functional morphology of locomotion requires a detailed understanding of appendicular anatomy. This is particularly true for the Crocodylia because they display a diversity of postures and gaits, from belly-sliding through to galloping [1–3], Consequently, anatomical and functional analyses of crocodilian musculature and locomotion have been widely used in muscle reconstructions of dinosaurs [4–16]. The locomotor variety apparent in both living and extinct members of the Crocodylia has promoted discussions on changes in musculoskeletal architecture of these clades [1,17–22].

To date, research into the appendicular morphology of Crocodylia has largely focused on the American alligator (*Alligator mississippiensis*). Many studies describe the limb anatomy of *A*. *mississipiensis* [11,14,20,23–25], with some comparing *A*. *mississipiensis* to a number of other crocodilian species [22], and individual species such as the South American *Caiman latirostris* [26], Southeast Asian *Crocodylus siamensis*, South American *Crocodylus acutus*, an unknown species of crocodile [27], and African *Osteolaemus tetraspis* [20]. In addition, kinematic studies have also been undertaken on the forelimb of *A*. *mississipiensis* to investigate mobility [3], and in the hindlimb to investigate postural changes between fossil and extant forms [1,3,21,28,29]. Currently no appendicular anatomical studies exist for *Crocodylus porosus* Schneider, 1801 and only a single study exists on the limb functional morphology of this species [30].

‘Digital dissection’ is a method which can aid in the interpretation and analysis of biological systems. Conducting a digital dissection is a non-destructive method that allows users to interact with data in three-dimensions. This gives researchers the ability to better visualise different muscles and identify their anatomical position. It further complements existing two-dimensional illustrations which are often simplified or stylised and hence open to misinterpretation [31–33]. Additionally, conducting a dissection digitally means anatomical features can be confirmed and re-examined [34]. Published digital dissections have been conducted on a variety of vertebrate taxa, largely focusing on cranial musculature [31,32,34–39], with a single paper using this technique to study pes myology in elephants [40]. Utilising computed tomography data (CT; predominately microCT) is the most common method used for digital dissections [34,38,39,41]. However, often CT does not provide enough soft tissue detail to conduct an adequate digital dissection [34]. To counteract this disadvantage contrast-enhancing agents like iodine have proven to be extremely useful for identifying detailed muscle architectures in small specimens [32,34,35,41–44]. For large specimens, passive diffusion of contrast-enhancing agents is not possible as penetration to muscles reduces greatly with size [43]. For this reason, digital dissection on larger specimens involves the use of a combination of CT and magnetic resonance imaging (MRI) data [39].

In the present study the use of both CT and MRI has enabled the formation of interactive three-dimensional models of the forelimb and hindlimb musculature of *C*. *porosus*. This is coupled with a detailed anatomical description to broaden the knowledge base for *C*. *porosus* and identify similarities and differences in appendicular anatomy between it and other crocodilian species.

## Materials

Two sub-adult male estuarine crocodile specimens were obtained from Koorana Crocodile Farm near Rockhampton, Queensland. They measured 2.1 metres (specimen number: XCb Cp4) and 1.6 metres (specimen number: XCb Cp5) in length. Specimens were killed by Koorana Crocodile Farm for their skins before being frozen for transport and use in scientific research. Body mass was not provided to us before skinning and freezing so we estimate mass of these specimens to be between 20 and 30 kilograms. Research was carried out under a Scientific Licence from the NSW National Parks and Wildlife Service, Office of Environment and Heritage (SL101730) to work on protected native fauna.

## Methods

### Digital dissection

Since it was not possible to use the iodine staining method in this study due to the size of the specimens, we chose to use both x-ray computed tomography (CT) and magnetic resonance imaging (MRI). The CT allowed us to visualise the bone in great detail, while the MRI provided greater muscle detail. CT and MRI scans were acquired for specimen XCb Cp4 at Armidale Radiology (Armidale NSW, Australia) after thawing.

CT scans were taken on a Siemens Syngo CT2012B. Scan data contained 3019 slices at 120KV and a slice thickness of 0.75mm. MRI scans were conducted on a GE Signa Excite, set to proton density weighted with a Tesla of 4. Forelimb and hindlimb scans were taken separately at a slice thickness of 4.0mm. Specimens were then refrozen at -4°C as a fresh-tissue dissection could not take place immediately after scanning.

**Fig 1:**
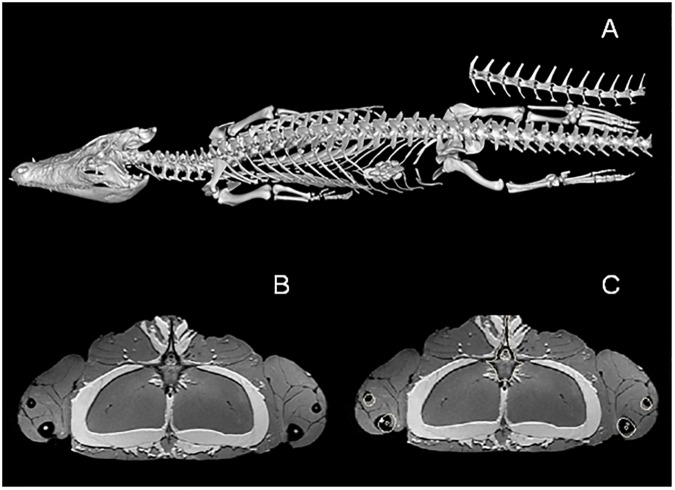
Combining CT and MRI data. CT and MRI data of *Crocodylus porosus* (XCb Cp4). A = skeleton extracted from CT; B = MRI coronal view of upper hindlimbs; C = same images as B with bone outlines from CT included.

MRI and CT data were analysed in Mimics 16.02 [45]. Skeletal information from the CT data was extracted using the ‘thresholding’ tool. Stl files of the forelimb and hindlimb skeleton were imported into the MRI files. The skeletal information was then overlaid on the MRI data as bone outlines (Fig 1). This made visualisation of the bone on the MRI scans much easier and subsequently improved the accuracy of the digital dissection. Muscles of both the forelimb and hindlimb were then manually segmented using the ‘Edit Mask’ tool in Mimics. It was only possible to segment out muscle bodies as resolution of the MRI scans was not high enough to also discern smaller structures like origin and insertion points. The digital dissection was carried out on the right forelimb and hindlimb and three-dimensional models were made of each muscle.

### Fresh-tissue dissection

A fresh-tissue dissection was performed on both specimens, beginning with XCb Cp4. This was completed to confirm MRI segmentation results and to complement and/or confirm features unclear or invisible on the MRI scan, particularly muscle-bone attachment sites. The combination of digital dissection and fresh-tissue dissection ensures the accuracy of differentiating closely associated muscles. For example, the muscles *deltoideus clavicularis*, *scapulohumeralis caudalis* and *teres major* all lie together on the lateral surface of the scapula blade. It was not possible to differentiate them from each other in the digital dissection, but they were easily identifiable in the fresh-tissue dissection. The dissection was conducted from superficial to deep muscle structures of both the forelimb and hindlimb.

### Muscle identification

**Fig 2:**
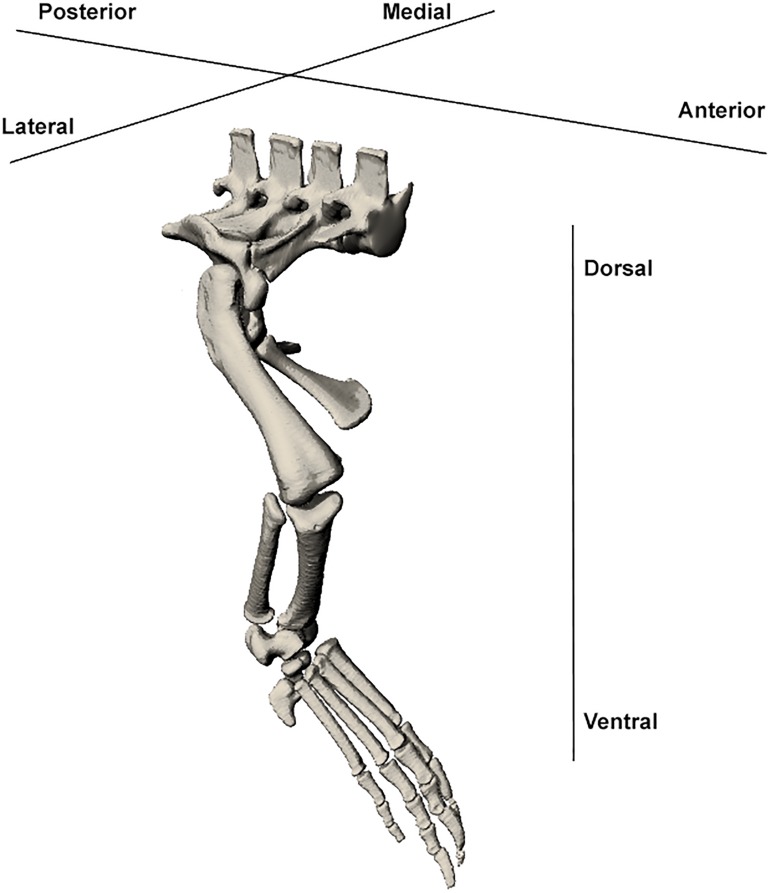
Orientation terminology used in muscle description. Diagram of the *Crocodylus porosus* hindlimb with terms used to describe orientation.

Identification of muscles was based on previously published limb descriptions of closely related species [11,17,20,22,23,26,27]. Due to some uncertainty surrounding the homology of archosaurian musculature, we have followed the most recent nomenclature used Reference 9 [9], which has been adopted by [26] and [22]. Mention has also been made of alternative nomenclature used [11,17,23,27,46] to assist with comparison between studies. Orientation terminology is outlined in Fig 2. Muscles were divided into groups. Forelimb muscles were split into extrinsic/intrinsic and forearm pronators/supinators. Intrinsic muscles were further split into extensor/flexor muscles of the shoulder, elbow and wrist [11,20]. Hindlimb muscles were divided into superficial/deep and then dorsal/ventral groups [11].

### Three-dimensional PDF

Data from both the digital and fresh-tissue dissections were combined to ensure accuracy of muscle location and origin/insertion points. To do this, muscles were exported from Mimics 16.02 and edited in the graphic design package ZBrush 4R7 [47]. Editing involved smoothing the muscles and extending muscle bodies to the attachment sites observed during the fresh-tissue dissection. Geometry of muscle bellies are largely retained, however muscle sizes should be treated as an approximation of cross-sectional area. Muscle-bone attachment sites were modelled in ZBrush based off the fresh-tissue dissection only, with notable tendons being modelled as separate structures. 3D models of each muscle or muscle division were assigned diverse colours to assist with differentiation and visualisation of muscles. The final result was then converted into two 3D PDFs for the forelimb and hindlimb following methods outlined by [33].

## Results

**Fig 3:**
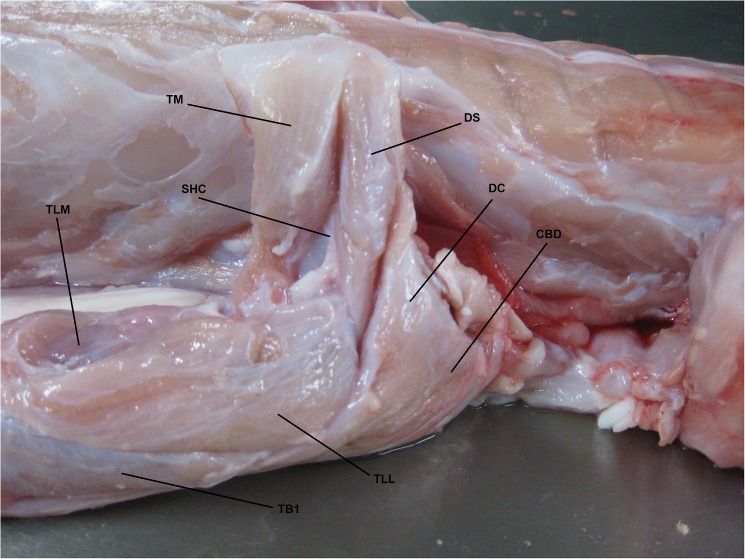
Fresh-tissue dissection with muscles identified around shoulder. Lateral view of right shoulder region of *Crocodylus porosus* (XCb Cp5). TM: *Teres major*, DS: *Deltoideus scapularis*, SHC: *Scapulohumeralis caudalis*, DC: *Deltoideus clavicularis*, CBD: *Coracobrachialis brevis dorsalis*, TLM: *Triceps longus medialis*, TLL: *Triceps longus lateralis*, TB1: *Triceps brevis 1*.

**Fig 4:**
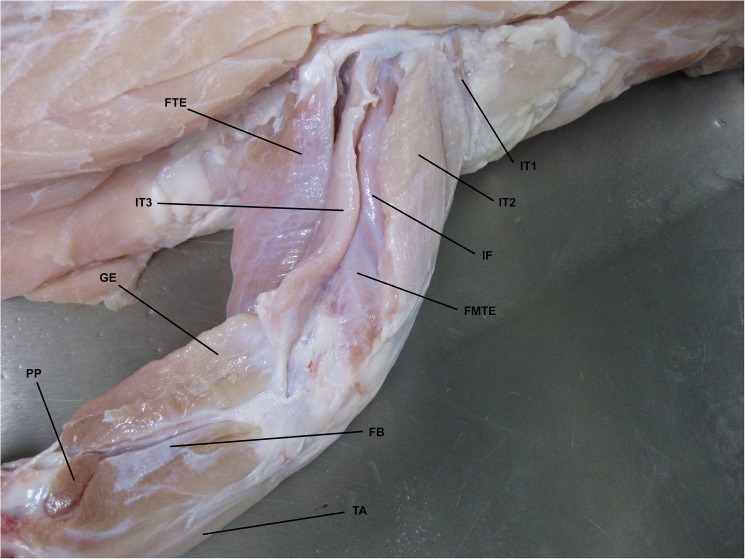
Fresh-tissue dissection with some hindlimb muscles identified. Dorsolateral view of right hindlimb of *Crocodylus porosus* (XCb Cp5). FTE: *flexor tibialis externus*, IT1-3: *Iliotibialis 1–3*, IF: *Iliofemoralis*, FMTE: *Femorotibialis externus*, GE: *Gastrocnemius externus*, PP: *Pronator profundus*, FB: *Fibularis brevis*, TA: *Tibialis anterior*.

The majority of muscles in both the forelimb and hindlimb of *C*. *porosus* could be correctly identified on the MRI scans based on their location, size and relationship to other muscles. However identifying the origin and insertion points of muscles was more difficult as they were often not visible on the MRI scans. Completing the fresh-tissue dissections allowed for the correct identification and documentation of origin/insertion points for most muscles, as well as an investigation into muscle function based on anatomy (Fig 3 and Fig 4). Function of some muscles described has also been measured using electromyographic (EMG) analysis [21,48]. Mention of functional similarities is included in the descriptions when applicable.

During the fresh-tissue dissection it became obvious that some of the large superficial dorsal muscles of the forelimb had been removed during the skinning process for both animals. It was therefore not possible to identify or model these muscles. These included the *trapezius*, *latissimus dorsi*, *rhomboideus* and *serratus ventralis cervicus*. A similar case was experienced in the lower limbs, where muscles inserting into the manus and pes had been severed during skinning. For this reason three muscles in the hindlimb could not be identified. These included the *flexor digitorum brevis*, *pronator quadratus* and *extensor hallucis longus*. Some muscles stretching into the manus and pes did retain their insertions even with skinning. Therefore we have included specific detail about insertions of those muscles, but for the muscles whose insertions were compromised we have left the description more general, simply describing insertion as “into the manus/pes”.

A detailed description of muscle anatomy, including comments on function, can be found below. Two 3D PDFs are also provided (S1 Fig and S2 Fig) as a visual and interactive guide to the muscle anatomy of both the forelimb and hindlimb of *C*. *porosus*.

### Forelimb (Fig 5; S1 Fig)

**Fig 5:**
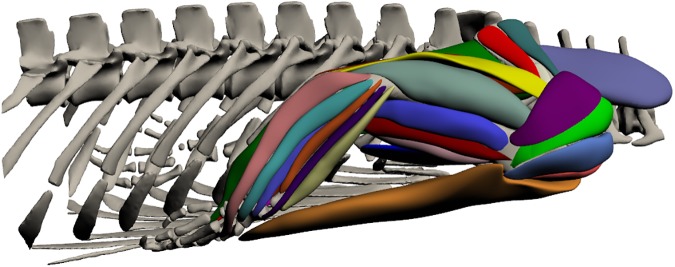
Three-dimensional model of forelimb musculature in *Crocodylus porosus*. Lateral view of the right forelimb. The interactive version of this model is available in ‘Supplementary Information’.

#### Superficial extrinsic muscles (Fig 6)

*Pectoralis* (PEC)

**Fig 6:**
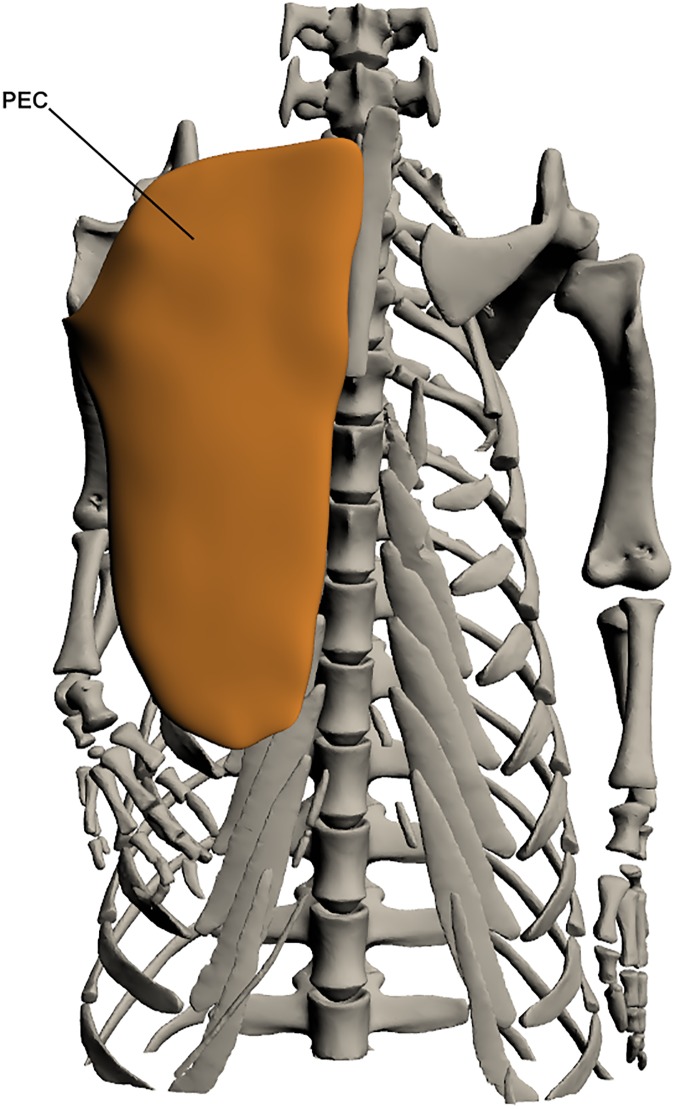
Superficial extrinsic muscles of the forelimb. PEC: *Pectoralis*.

This muscle originates by a fleshy attachment along the ventral midline of the animal from the interclavicle and sternal ribs. It inserts by a tendon on the proximo-ventral humerus at the deltopectoral crest. Its principle function is in shoulder and limb adduction. It is a very large sheet-like muscle that covers the entire superficial ventral chest area.

#### Deep extrinsic muscles (Fig 7)

*Levator scapulae* (LS) (*collo-scapularis supericialis* [27])

**Fig 7:**
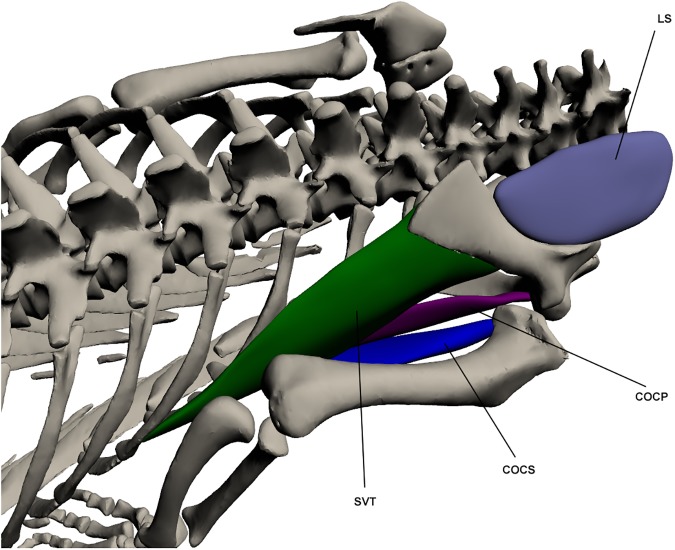
Deep extrinsic muscles of the forelimb. LS: *Levator scapulae*, SVT: *Serratus ventralis thoracis*, COCS: *Costocoracoideus superficialis*, COCP: C*ostocoracoideus profundus*.

This muscle’s origin lies with the neck muscles on the lateral cervical ribs. It inserts on the anterior border of the scapula by a fleshy attachment which extends from the most antero-distal part of the scapula to the glenohumeral joint and is largely obscured from view by the *trapezius* which overlies this part of the LS [20]. It plays a major part in drawing the shoulder anteriorly.

*Serratus ventralis thoracis* (SVT) (*serratus posterior* [23]; *thoracic-scapularis superficialis* [27])

This muscle originates from the trunk at the ventro-lateral ribs. It inserts by a tendon on the entire medio-posterior margin of the scapula. It is a broad but thin muscle involved in extension of the pectoral girdle.

*Costocoracoideus profundus* (COCP)

This muscle originates in the trunk on the lateral margin of the first few ribs and inserts at the ventro-posterior margin of the coracoid. Both attachment points are fleshy. This muscle is long but thin and lies deep to the *pectoralis*. It functions as a pectoral girdle extensor.

*Costocoracoideus superficialis* (COCS)

This muscle originates on the ventro-lateral first few ribs by a fleshy attachment and inserts onto the medial humeral head, also by a fleshy attachment. It lies under the pectoralis and ventral to the *costocoracoideus profundus*, and is broad at origin becoming narrower but thicker towards insertion. It has a minor role in extension of the shoulder girdle.

### Intrinsic muscles

#### Shoulder extensors/flexors (Fig 8)

*Teres major* (TM)

**Fig 8:**
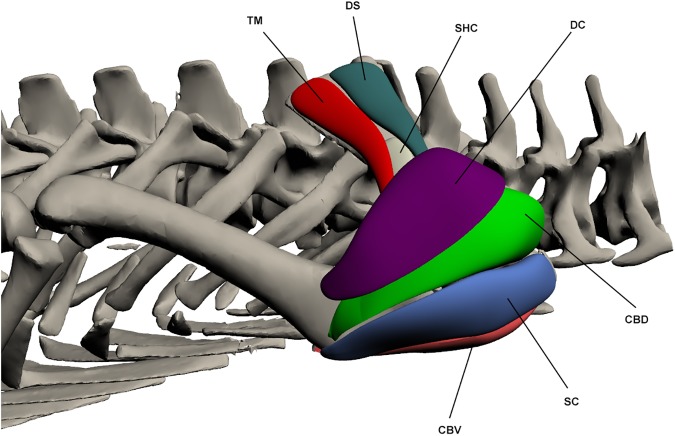
Intrinsic muscles of the forelimb: shoulder extensors and flexors. TM: *Teres major*, DS: *Deltoideus scapularis*, SHC: *Scapulohumeralis caudalis*, DC: *Deltoideus clavicularis*, CBV: *Coracobrachialis brevis ventralis*, SC: *Supracoracoideus*, CBD: *Coracobrachialis brevis dorsalis*.

This muscle originates on the disto-lateral surface of the scapula by a fleshy attachment, and inserts via a long and well-defined tendon onto a bony ridge on the proximo-lateral humerus. TM makes up the postero-lateral surface of the scapula and lies deep to the *latissimus dorsi*. It is involved in shoulder abduction, and also flexion to a minor degree.

*Deltoideus scapularis* (DS) (*scapular triceps* [11])

This muscle originates by a fleshy attachment on the disto-lateral scapula and inserts by a tendon on the dorso-lateral humeral head. It is an elongate muscle that attaches to the scapula along its length from point of origin until it becomes tendinous. It lies anterior to *teres major*. The DS acts as a shoulder abductor and flexor.

*Supracoracoideus* (SC)

This muscle originates at the proximo-lateral coracoid by a fleshy attachment and covers the entire humeral head. It inserts on the ventro-lateral humerus on the deltopectoral crest by a fleshy attachment. The SC is very broad and triangular in shape, making up much of the shoulder. It acts as a shoulder extensor and, to a minor degree, adductor.

*Scapulohumeralis caudalis* (SHC) (*teres minor* [11,23])

This muscle originates on the proximo-lateral and posterior scapula, and inserts on the proximo-lateral humeral head. Both attachments are fleshy. It is a small muscle that is largely overlain by *teres major* posteriorly and *deltoideus scapularis* anteriorly. The SHC functions as a shoulder abductor.

*Coracobrachialis brevis ventralis* (CBV)

This muscle originates by a fleshy attachment over the entire lateral coracoid and inserts, also by a fleshy attachment, onto the deltopectoral crest. It lies deep to the *pectoralis* and posterior to the *biceps brachii*. It is a large and broad muscle that covers the lateral coracoid and proximo-ventral humerus. The CBV acts as a shoulder and limb adductor.

*Coracobrachialis brevis dorsalis* (CBD)

This muscle originates from the antero-lateral and proximal scapula by a fleshy attachment. It inserts on the proximal third of the anterior humerus near the deltopectoral crest. This muscle is triangular in shape and makes up much of the bulk of the shoulder along with the *supracoracoideus*. It acts as a shoulder extensor and possibly also as a joint stabiliser [20].

*Subscapularis* (SS)

This muscle originates on the postero-lateral scapula by a fleshy attachment and inserts by a fleshy attachment on the dorso-lateral humeral head. It is a shoulder extensor and has also been identified as a shoulder stabiliser due to its small size and limited mechanical advantage [20].

*Deltoideus clavicularis* (DC) (*scapular deltoid* [11])

This muscle originates by a fleshy attachment on the antero-lateral scapula and inserts on the dorso-lateral head of the humerus, again by a fleshy attachment. It makes up much of the bulk of the shoulder along with the *supracoracoideus*, overlying most of *coracobrachialis brevis dorsalis* as well as the insertion of *deltoideus scapularis*. It can sometimes be difficult to differentiate from *coracobrachialis brevis dorsalis* and *supracoracoideus*. The DC acts to extend the shoulder.

#### Elbow extensors/flexors (Fig 9)

*Humeroradialis* (HR) (*brachio-radialis* [49])

**Fig 9:**
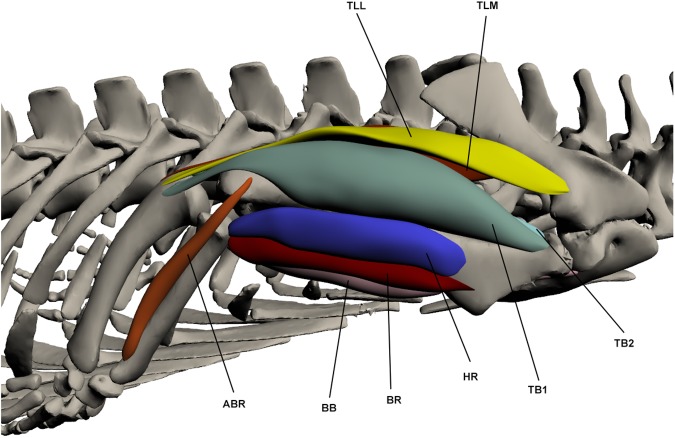
Intrinsic muscles of the forelimb: elbow extensors and flexors. TLL: *Triceps longus lateralis*, TLM: *Triceps longus medialis*, ABR: *Abductor radialis*, BB: *Biceps brachii*, BR: *Brachialis*, HR: *Humeroradialis*, TB1-2: *Triceps brevis 1–2*.

This muscle originates on the ventro-lateral humerus at the deltopectoral crest by a fleshy attachment. It inserts with *biceps brachii* and *brachialis* on the proximo-medial radius by a short tendon. The HR lies anterior to the *biceps brachii* and *brachialis*; it is also broader than these other two muscles. It functions to flex the elbow.

*Biceps brachii* (BB) (*coracoantebrachialis* [11,23])

This muscle originates on the ventral coracoid by a very long tendon (half the length of the muscle belly). It inserts by a tendon onto the proximo-medial radius. This muscle is rather slender and is a major elbow flexor.

*Brachialis* (BR)

This muscle originates by a fleshy attachment on the anterior humeral head. It inserts onto the proximo-medial radius with the tendon of the *biceps brachii*. The BR is a thin and elongate muscle that attaches to the humerus along its length. It lies between the *biceps brachii* and the *humeroradialis*. It is involved in elbow flexion.

*Triceps longus lateralis* (TLL) (*anconeus scapularis* [11,23])

This muscle originates at the proximo-lateral scapula via a tendon, superficial to the *teres major* and *scapulohumeralis caudalis*, but deep to *deltoideus scapularis*. It inserts by a tendon onto the lateral olecranon process of the proximal ulna. It makes up much of the dorsal surface of the upper arm and extends over the medial surface of *triceps brevis 1*. This muscle’s primary function is in elbow extension.

*Triceps longus medialis* (TLM) (*anconeus scapulocoracoideus* [11,23])

This muscle originates by a tendon from the postero-lateral and proximal surface of the scapula, deep to the *teres major*, and inserts by a tendon on the olecranon process of the proximal ulna with the *triceps longus lateralis*. TLM lies posterior to the *triceps longus lateralis* and is of a similar size and shape. It is an elbow extensor.

*Triceps brevis* (TB) (TB1 = *Anconeus humeralis lateralis*, TB2 = *anconeus humeralis medials*, TB3 = *anconeus major* [11,23])

This muscle is composed of 3 parts. TB1 originates by a fleshy attachment on the proximo-lateral humerus, overlying the insertion of *teres major*. It inserts by a tendon on the proximal ulna at the olecranon process and attaches to the humerus for part of its length. TB2 originates on the postero-dorsal and proximal humerus, while TB3 originates on the posterior humeral head. TB2 and TB3 insert together with TB1 also by a tendinous attachment. TB2 and TB3 run postero-dorsally along the humerus and sit flush against the bone, attaching along the length of the muscle. TB1 overlies much of TB2. All parts of this muscle are involved in elbow extension.

*Abductor radialis* (ABR) (*humero-radialis internus* [11,23])

This muscle originates by a tendon from the disto-lateral humerus and inserts by a fleshy attachment on the distal lateral radius. It runs along the lateral surface of the radius and attaches along its length. It lies between the *extensor carpi radialis longus* and *extensor carpi radialis brevis*. The ABR is an elbow flexor.

#### Wrist extensors/flexors (Fig 10)

*Flexor carpi ulnaris* (FCU) (*humero-radialis lateralis* [23,27])

**Fig 10:**
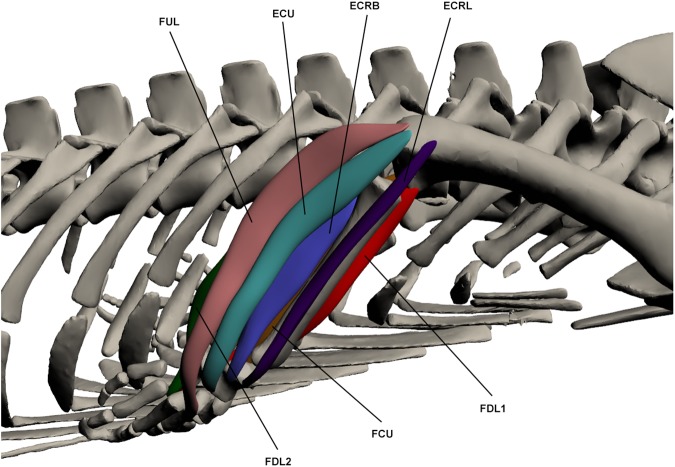
Intrinsic muscles of the forelimb: wrist extensors/flexors. FUL: *Flexor ulnaris longus*, ECU: *Extensor carpi ulnaris*, ECRB: *Extensor carpi radialis brevis*, ECRL: *Extensor carpi radialis longus*, FDL1-2: *Flexor digitorum longus*, FCU: *Flexor carpi ulnaris*.

This muscle originates via a tendon at the disto-medial humerus and inserts by a fleshy attachment at the ventral manus. It lies medial to *flexor digitorum longus 1* and is a relatively slender muscle. It is one of the major wrist flexors.

*Flexor digitorum longus* (FDL) (*flexor digitorum communis profundus* [23])

This muscle is composed of 2 parts. FDL1 originates on the ventro-medial and distal humerus via a tendinous attachment and inserts by a tendinous attachment into the manus at digit 2 with FDL2. It lies between *flexor carpi ulnaris* medially and *pronator teres* laterally, and is a relatively thin muscle. FDL2 originates by a fleshy attachment at the proximo-medial head of the ulna before inserting via a tendon into the manus on the ventral surface of digits 1–3. FDL2 attaches to the ulna along its length. It is a thin muscle at its origin point but broadens substantially at the distal ulna before thinning again and becoming tendinous. Both parts of this muscle are involved in wrist flexion.

*Extensor carpi radialis longus* (ECRL) (*extensor carpi radialis* [23])

This muscle originates on the ventro-lateral humerus by a tendinous attachment and inserts, also by a tendon, on the dorsal manus. It is a long and relatively thin muscle that lies anterior to the *abductor radialis*. The ECRL functions as a wrist extensor.

*Extensor carpi radialis brevis* (ECRB) (*extensor carpi radialis profundus* [23]*; supinator manus* [46])

This muscle originates with two heads which are almost indistinguishable, one on the antero-proximal head of the ulna and the other on the poster-proximal radius, both by fleshy attachments. ECRB then inserts at the dorsal manus via a tendon. The origin of this muscle lies deep to the *extensor carpus ulnaris* and *abductor radialis*. It is involved in wrist extension.

*Extensor carpi ulnaris* (ECU) (*humero-carpi ulnaris* [23]*; ectepicondylo-ulnaris* [46])

This muscle originates by a tendon on the disto-ventral humerus at the lateral condyle and inserts at the dorsal manus via a tendon. It acts as an elbow flexor and wrist extensor.

*Flexor ulnaris longus* (FUL) (*humerodorsalis* [23])

This muscle originates at the disto-lateral humerus via a tendinous attachment. It runs along the lateral ulna and inserts by a tendon onto the dorsal metacarpals. This muscle is wider at origin and thins towards its insertion point. It lies posterior to the *extensor carpi ulnaris*. The FUL functions as an elbow flexor and wrist extensor.

#### Pronators/supinators of the forearm (Fig 11)

*Pronator teres* (PT) (*ulno-radialis* [23])

**Fig 11:**
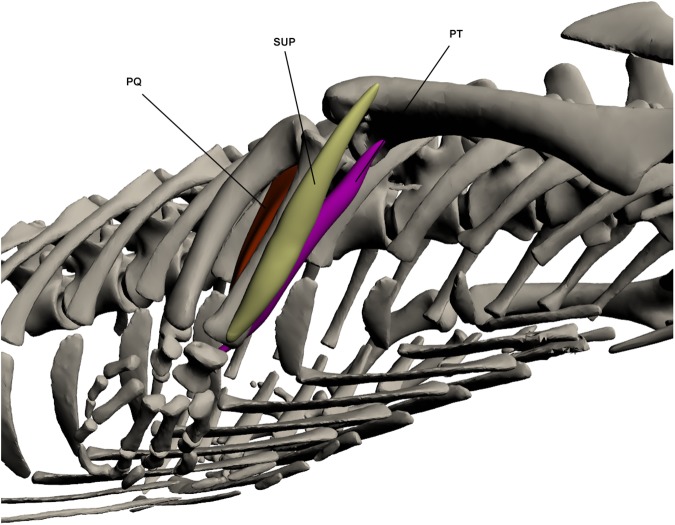
Intrinsic muscles of the forelimb: pronators and supinators of the forearm. PQ: *Pronator quadratus*, SUP: *Supinator*, PT: *Pronator teres*.

This muscle originates by a tendinous attachment at the ventro-distal humerus and inserts by a fleshy attachment on the ventral carpals. It attaches to the antero-medial radius along its length and lies between *supinator* anteriorly and *flexor digitorum longus 1* posteriorly. Its principal function is in wrist pronation.

*Pronator quadratus* (PQ)

This muscle originates by a fleshy attachment at the proximo-medial ulna and inserts on the postero-distal radius. PQ is very broad at origin but thins towards its insertion point. It lies deep to the other ventral muscles of the forearm, between the radius and ulna. It is an elbow pronator and also assists in stabilising the radius and ulna [20].

*Supinator* (SUP)

This muscle originates by a tendinous attachment at the disto-lateral humerus and runs along the lateral radius, attaching to the bone, inserting by a tendon on the antero-distal radius. It partially overlies *extensor carpi radialis longus* at origin. SUP is a large muscle that lies on the anterior edge of the radius. It acts as the major wrist supinator.

### Hindlimb (Fig 12; S2 Fig)

**Fig 12:**
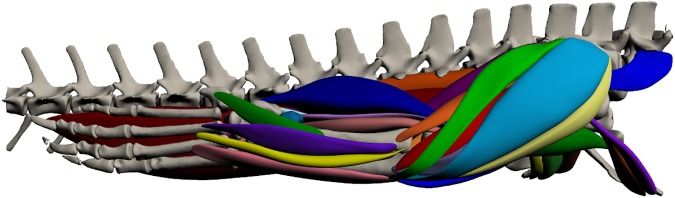
Three-dimensional model of hindlimb musculature in *Crocodylus porosus*. Lateral view of the right hindlimb. The interactive version of this model is available in ‘Supplementary Information’.

#### Superficial dorsal muscles of the upper hindlimb (Fig 13)

*Iliotibialis* (IT) (*extensor iliotibialis* [23,27])

**Fig 13:**
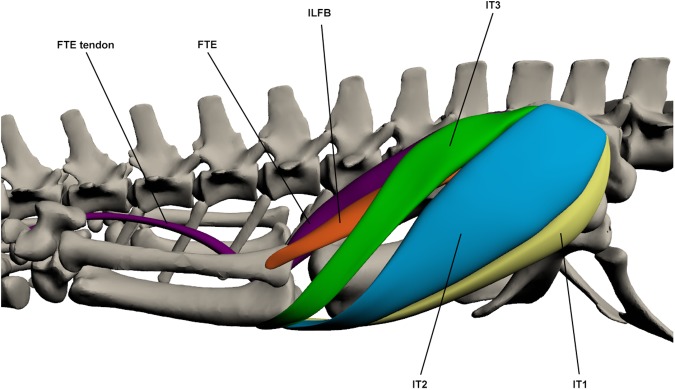
Superficial dorsal muscles of the upper hindlimb. ILFB: *Iliofibularis*, FTE: *Flexor tibialis externus*, IT1-3: *Iliotibialis 1–3*.

This muscle is made up of 3 parts. IT1 originates by a fleshy attachment from the antero-lateral ilium and inserts via a tendinous attachment to the proximo-anterior tibia, inserting with IT2 and IT3. It is a relatively slender muscle. IT2 originates by a tendon from the central lateral ilium between IT1 and IT3. It inserts on the proximo-anterior tibia via a tendinous attachment. It is a sheet-like muscle and is the largest of the *iliotibialis* muscles, making up almost the complete dorsolateral face of the thigh. It is broad at the proximal end, thinning and narrowing towards its insertion. IT3 originates by a fleshy attachment on the postero-lateral ilium and inserts on the proximo-anterior tibia with IT2, also by a tendon. It lies directly posterior to IT2 and anterior of *flexor tibialis externus*. It is of a similar shape to IT1. The *iliotibialis* muscles make up the entire of the superficial dorso-lateral thigh are all used in extension of the knee. Knee extensor function of IT2 is supported by EMG data [21,48].

*Flexor tibialis externus* (FTE)

This muscle originates by a tendon from the postero-lateral ilium, posterior to *iliotibialis 3*, and inserts via a long tendon, shared with *gastrocnemius externus*, into the pes. The muscle body extends to the insertion of the *flexor tibialis internus* muscles at the knee, beyond that the tendon continues by stretching medial to the *gastrocnemius externus* to the final insertion point at the proximal pes. The FTE is a large muscle that covers the posterior surface of the thigh and acts as a hip extensor and knee flexor. Hip extension function is supported by EMG data [21,48].

*Iliofibularis* (ILFB)

This muscle originates by a fleshy attachment at the central lateral ilium ventral to the origin of *iliotibialis 3*. It inserts on the proximo-lateral fibula via a tendinous attachment. The distal part of the muscle belly is visible between *iliotibialis 3* and *flexor tibialis externus*. It is a thin muscle that acts as a hip extensor and knee flexor. Knee flexion function is supported by EMG data, which also suggests function as a hip abductor [48].

#### Superficial ventral muscles of the upper hindlimb (Fig 14)

*Ambiens* (AMB) (*pubo-femoralis* [23]; *sartorius* [17])

**Fig 14:**
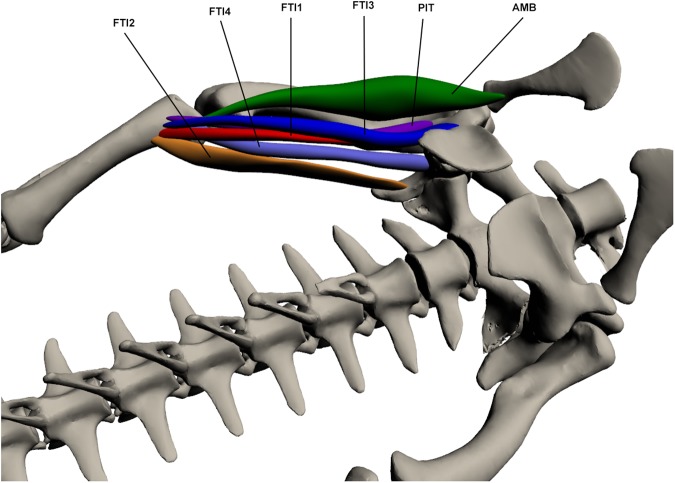
Superficial ventral muscles of the upper hindlimb. AMB: *Ambiens*, PIT: *Pubo-ischio-tibialis*, FTI1-3: *Flexor tibialis internus 1–3*.

This muscle originates on the proximo-lateral pubis by a tendinous attachment. It inserts on the proximo-anterior tibia at the cnemial crest via a long tendon. This muscle is wide at origin, thinning as it becomes tendinous for insertion. It is visible along the anterior surface of the thigh. Only a single head was identified in *C*. *porosus*. It is a knee extensor and hip flexor. Knee extension function is supported by EMG data [21,48].

*Flexor tibialis internus* (FTI)

This muscle is composed of 4 parts. FTI1 originates from the postero-lateral ischium and inserts on the proximo-medial tibia via a long tendon with *pubo-ischio-tibialis*. It is a long and thin muscle. FTI2 originates by a fleshy attachment on the postero-ventral ilium and inserts on the proximo-medial tibia via a tendon with FTI1. It is the largest of the 4 parts and is broad. FTI3 originates on the proximo-lateral ischium and inserts by a tendon on the proximo-medial tibia with the other FTI muscles. It is broader than FTI1 and lies ventral to the secondary tendon of the *caudofemoralis longus*. FTI4 originates on the latero-ventral ilium near the ischium and inserts via a tendon with the other three FTI muscles. It is a small thin muscle. The FTI muscles are involved in hip extension and knee flexion. Hip extension function in FTI2 is supported by EMG data [48].

*Pubo-ischio-tibialis* (PIT)

This muscle originates by a tendon at the postero-lateral ischium near where the ischium and pubis join, and inserts by a tendon on the proximo-medial tibia. It is a long and relatively thin muscle that lies lateral to *flexor tibialis internus 1*. Its origin also lies between *pubo-ischio-femoralis externus 3* and *adductor 1*. The PIT is involved in hip extension and knee flexion; it also functions as an adductor to a minor degree. Knee flexion and hip adduction function is supported by EMG data [21,48].

#### Deep dorsal muscles of the upper hindlimb (Fig 15)

*Iliofemoralis* (IF) (*caudali-ilio-femoralis* [23])

**Fig 15:**
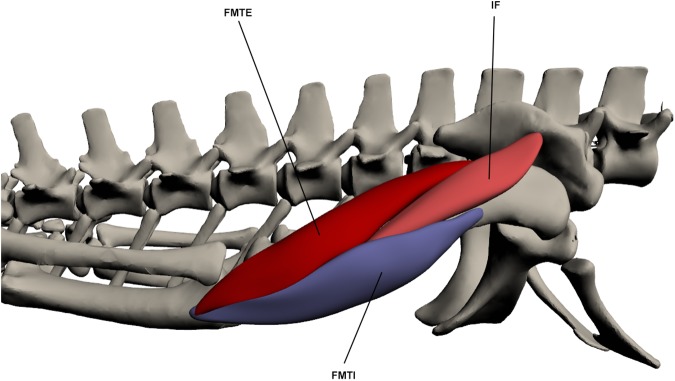
Deep dorsal muscles of the upper hindlimb. FMTE: *Femorotibialis externus*, IF: *Iliofemoralis*, FMTI: *Femorotibialis internus*.

This muscle originates deep to *iliotibialis 2* on the central lateral ilium via a tendinous attachment. It inserts by a fleshy attachment onto the distal third of the dorso-lateral femur between *femorotibialis internus* and *femorotibialis externus*. The IF is tear shaped and attaches along most of the length of the femur. It is one of the hip abductor muscles. Hip abduction function is supported by EMG data [21,48].

*Femorotibialis internus* (FMTI)

This muscle originates by a tendon on the dorso-proximal femur, distal and anterior to *iliofemoralis*. It then inserts on the cnemial crest via a tendon with *femorotibialis externus*. FMTI attaches to the femur and envelops the dorso-lateral portion of this bone. It is involved in knee extension. Knee extension function is supported by EMG data [21,48].

*Femorotibialis externus* (FMTE)

This muscle originates by a fleshy attachment approximately one third of the way down the dorsal femur. It inserts on the proximo-lateral cnemial crest by a small tendon. FMTE merges with *femorotibialis internus* by a tendon at its insertion point but is separated at origin by the *iliofemoralis*. It lies posterior to *femorotibialis internus* and is smaller than this muscle. It functions as a knee extensor.

#### Deep ventral muscles of the upper hindlimb (Fig 16)

*Caudofemoralis longus* (CFL) (*coccygeo-femoralis longus* [17]; *caudi-femoralis* [27])

**Fig 16:**
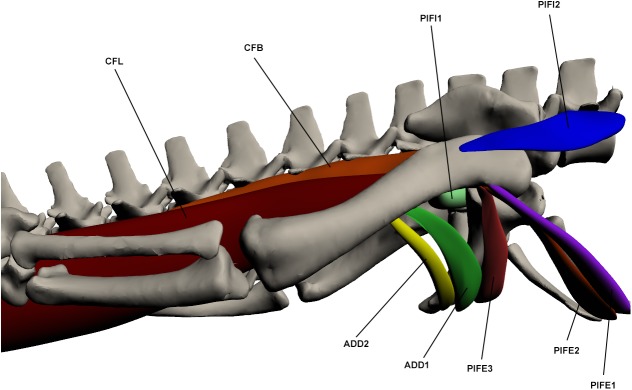
Deep ventral muscles of the upper hindlimb. CFL: *Caudofemoralis longus*, CFB: *Caudofemoralis brevis*, PIFI1-2: *Pubo-ischio-femoralis internus 1–2*, ADD 1–2: *Adductor 1–2*, PIFE 2–3: *Pubo-ischio-femoralis externus 2–3*.

This muscle originates by a fleshy attachment in the tail at numerous caudal vertebral haemal arches beginning at caudal vertebra 12. It inserts via a very large tendon on the proximo-ventral femur at the fourth trochanter. This muscle also has an accessory tendon that attaches at the ventro-distal femur on the tibial condyle with the *gastrocnemius externus*. This accessory tendon is covered in dorsal view by *iliotibialis 3* and *flexor tibialis externus*. The proximal third of this muscle, between its origin and insertion points, sits free from the caudal vertebrae and is therefore constrained by the hypaxial musculature, namely the *transverse perenei*, and also a layer of fat that separates the CFL from the hypaxial muscles. CFL is the largest muscle in the crocodile’s body and performs the majority of the hip extension along with c*audofemoralis brevis*. Hip extension function is supported by EMG data, which also suggests function as a femoral mediolateral rotator [48].

*Caudofemoralis brevis* (CFB) (*coccygeo-femoralis brevis* [17,23]; *caudi-femoralis* [27])

This muscle originates in the tail by two heads, one from the postero-lateral ilium and the other to caudal vertebrae 4 and 5, anterior to the origin of *caudofemoralis longus*. It inserts by a tendon at the proximo-ventral femur on the fourth trochanter. It lies antero-medial to the *caudofemoralis longus* and is the smaller of the two major hip extensors.

*Adductor* (ADD) (*adductor femoris* [17,23]; *ischio-femoralis* [27])

This muscle is composed of 2 parts. ADD1 originates by a fleshy attachment at the ventro-lateral surface of the ischium and inserts via a tendon at the at the ventro-medial femur. ADD2 lies posterior to ADD1. It also originates by a fleshy attachment on the ventro-lateral ischium and inserts on the disto-ventral femur again by a fleshy attachment. It is much longer and thinner than ADD1. The adductor is important for hip adduction and also contributes to extension of the hip joint. Hip adduction function in ADD1 is supported by EMG data [21,48].

*Pubo-ischio-femoralis externus* (PIFE)

This muscle is composed of 3 parts. PIFE1 originates on the anterior boarder of the pubis, anterior to PIFE2, by a fleshy attachment. It inserts on the proximo-anterior femur with the other two parts of PIFE. PIFE2 originates from the entire lateral pubis and inserts via a tendon at the proximo-anterior femur. It is a fan-shaped muscle. PIFE3 originates by a fleshy attachment on the antero-lateral margin of the ventral ischium and inserts by a tendon on the proximo-anterior femur with PIFE2. It is less broad than PIFE2 but is thicker. PIFE functions as a hip adductor. Hip flexion function in PIFE2 and PIFE3 is supported by EMG data, which also suggests function as a hip adductor [21,48].

*Pubo-ischio-femoralis internus* (PIFI) (*quadratus lumborum* [23])

This muscle is composed of 2 parts. PIFI1 originates on the medial ilium and proximo-medial ischium by a fleshy attachment, and inserts on the proximal femur, medial to the fourth trochanter and between the insertion of *pubo-ischio-femoralis externus 3* and *adductor 1*. It is a short but thick muscle. PIFI2 originates anterior to the pelvic girdle by a fleshy attachment on the transverse processes of the few vertebrae anterior to the girdle. It inserts by a tendon onto the proximo-lateral femur near the fourth trochanter. It is a fan-shaped muscle. PIFI1 acts as a hip adductor while PIFI2 acts to both abduct and flex the hip. Hip flexion function in PIFI2 is supported by EMG data, however this data also suggests a hip adductor function, as in PIFI1, rather than abduction [21,48].

#### Superficial dorsal muscles of the lower hindlimb (Fig 17)

*Gastrocnemius externus* (GE)

**Fig 17:**
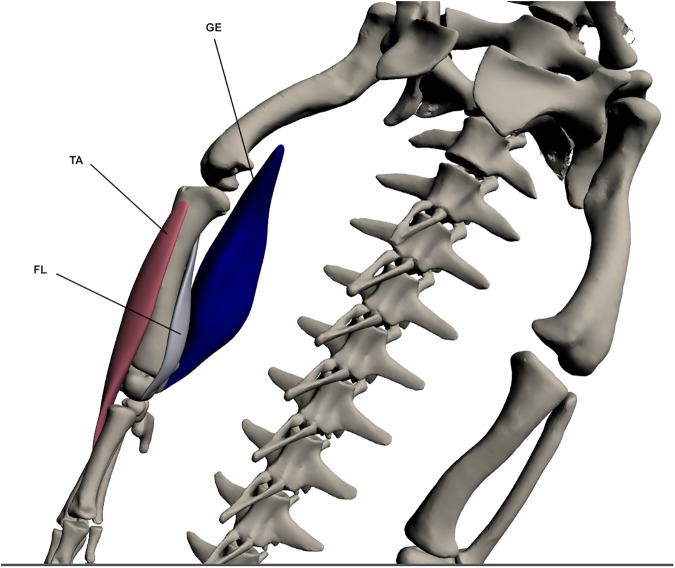
Superficial dorsal muscles of the lower hindlimb. GE: *Gastrocnemius externus*, TA: *Tibialis anterior*, FL: *Fibularis longus*.

This muscle originates by a fleshy attachment at the *caudofemoralis longus* tendon on the postero-distal femur. It inserts by a wide tendon onto the lateral calcaneus. It is a sizeable muscle, making up a large portion of the postero-lateral lower limb. It is largely responsible for extension of the knee.

*Tibialis anterior* (TA) (*tibialis anticus* [23,27])

This muscle originates via a fleshy attachment on the antero-proximal tibia. Insertion is by a tendon into the dorsal surface of digits 2 and 3. It is a large and elongate muscle covering the anterior tibia and is involved in ankle flexion.

*Fibularis longus* (FL)

This muscle’s origin is on the proximo-medial tibia by a tendon and it inserts by a tendon at the astragalus. The FL lies postero-medial to *tibialis anterior*. It acts to flex the ankle.

#### Superficial ventral muscles of the lower hindlimb (Fig 18)

*Extensor digitorum longus* (EDL) (*extensor digiti* [27])

**Fig 18:**
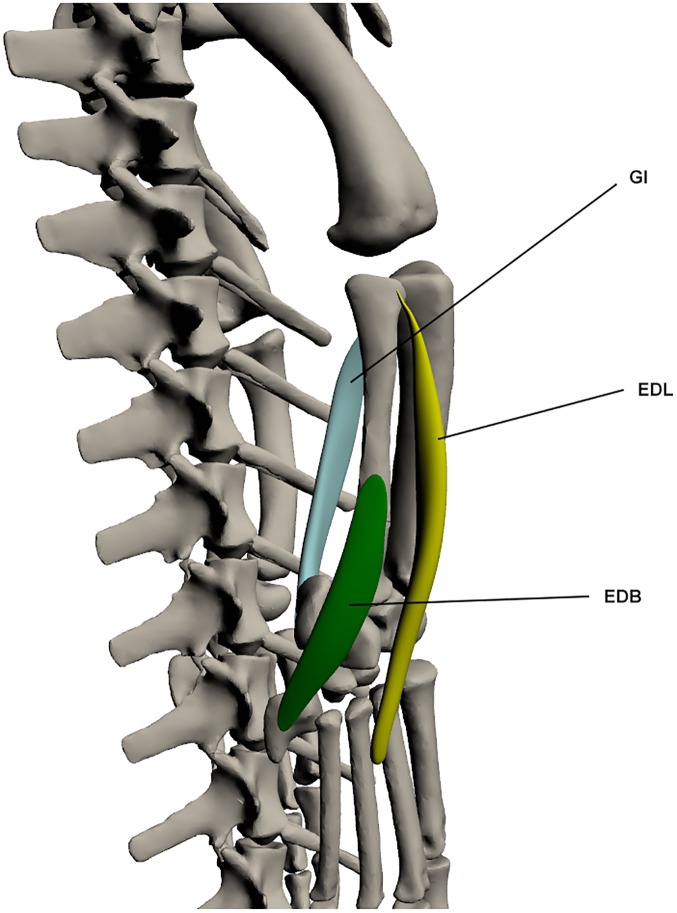
Superficial ventral muscles of the lower hindlimb. GI: *Gastrocnemius internus*, EDL: *Extensor digitorum longus*, EDB: *Extensor digitorum brevis*.

This muscle originates as a narrow tendon on the lateral fibula, and inserts onto the dorsal surface of the digits via a tendinous attachment. It lies postero-lateral to *tibialis anterior*. The EDL is involved in ankle extension. It also functions as a digit extensor.

*Extensor digitorum brevis* (EDB) (*peroneus posterior* [27])

This muscle originates by a fleshy attachment at the latero-distal fibula and inserts into the dorsal pes at digit 5. It is a small but broad muscle and lies distal and posterior to the *extensor digitorum longus* and *fibularis brevis*. EDB functions to extend the pes.

*Gastrocnemius internus* (GI)

This muscle originates by a tendon from the proximo-medial tibia and inserts at the ventral calcaneus by a tendon with the *gastrocnemius externus*. It is wide but thin and lies deep to the *gastrocnemius externus*. GI is one of the ankle extensors.

#### Deep dorsal muscles of the lower hindlimb (Fig 19)

*Flexor digitorum longus* (FDL)

**Fig 19:**
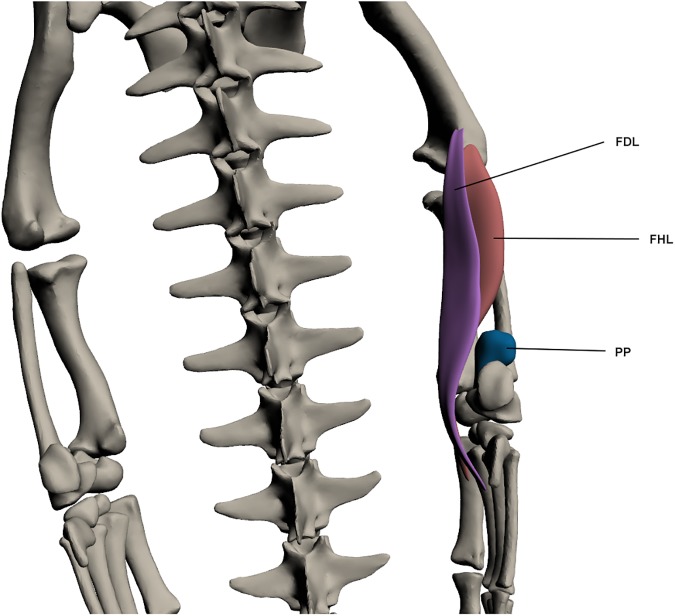
Deep dorsal muscles of the lower hindlimb. FDL: *Flexor digitorum longus*, FHL: *Flexor hallucis longus*, PP: *Pronator profundus*.

This muscle originates by a tendon on the ventro-lateral femur at the lateral condyle and inserts into the ventral pes by a tendon. This muscle is long and wide but quite thin. It lies directly over the *flexor hallucis longus* and is a knee extensor and also a digit flexor.

*Flexor hallucis longus* (FHL) (*tibialis posticus* [27])

This muscle originates by a fleshy attachment from the lateral condyle of the femur and inserts by a tendon past the knee and into the ventral pes. It is wide at the proximal end and tapers towards insertion. It covers much of the postero-ventral fibula and functions to extend the knee and flex the digits.

*Pronator profundus* (PP)

This muscle originates from the disto-lateral fibula via a fleshy attachment. It inserts at the tarsals, again by a fleshy attachment. PP is a small and almost spherical muscle on the disto-lateral fibula. It is involved in ankle extension, while its ability to pronate the ankle seems to be limited by its small size.

#### Deep ventral muscles of the lower hindlimb (Fig 20)

*Fibularis brevis* (FB) (*peroneus anterior* [23])

**Fig 20:**
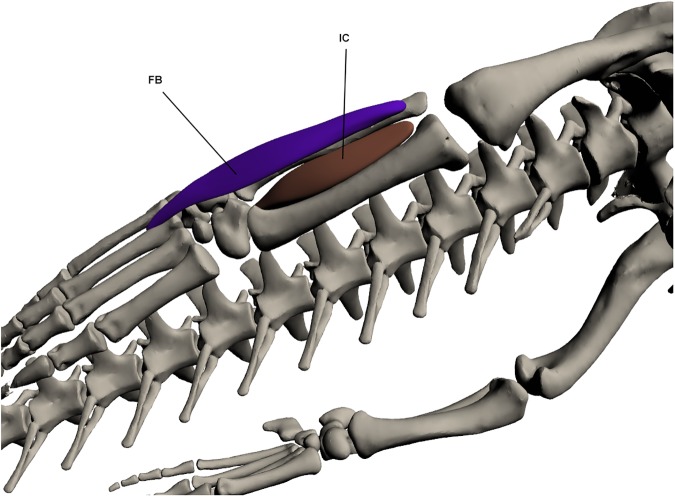
Deep ventral muscles of the lower hindlimb. FB: *Fibularis brevis*, IC: *Interosseus cruris*.

This muscle originates on the lateral fibula by a fleshy attachment and inserts via a tendon on the dorsal surface of metatarsal 3. This muscle is narrow at origin but broadens towards insertion before becoming tendinous. It lies posterior to *extensor digitorum longus*. It functions to flex the ankle joint.

*Interosseus cruris* (IC)

This muscle originates by a tendon at the proximo-medial tibia and fibula. It inserts via a tendon into the ventral pes. It lies between the tibia and fibula and fills the space between these two bones. It functions to assist in ankle joint flexion.

## Discussion

The dissections conducted here revealed differences in muscle anatomy between *Crocodylus porosus* and other crocodilians. The general forelimb morphology of crocodilians is conservative [20,46] compared to the hindlimb which has experienced many more changes thought to reflect the evolution of locomotion in the Archosauria [2,7]. Most likely for this reason, more variation was identified in the hindlimb than in the forelimb. Variations noted include changes to the number of heads of a muscle, and the type and location of origin and insertion.

The number and arrangement of muscles in the *flexor cruris* group (FTI1-4, PIT and FTE) has differed in previous studies. Three parts are found in *A*. *mississippiensis* and *C*. *latrostris* [11,23,26,27], as opposed to the four parts described in other studies [9,14,17]. Here four parts were described for *C*. *porosus*. We found that the *ambiens* muscle in *C*. *porosus* was only composed of a single part as opposed to the two parts found in *A*. *mississippiensis* [9,17,27]. This finding concurs with that of a previous study [26] that also only found a single head to the *ambiens*. They suggest this could be the result of a retained plesiomorphic condition or due to the sub-adult nature of the specimens they were examining. Due to the phylogenetic distances between the *Caiman* and *Crocodylus* [50], we propose the presence of only a single head to the *ambiens* is the result of obtaining sub-adult specimens in both studies. In the lower hindlimb, some descriptions on *A*. *mississippiensis* mention two parts to the *flexor digitorum longus* muscle [14,23], whereas only a single part has been identified here.

In studies on *A*. *mississippiensis* [17], *C*. *latirostris* [26] and an unknown species of crocodile [27], a secondary tendon has been described for the *iliofibularis* which comes from the main body of this muscle and inserts with the *gastrocnemius internus*. This tendon was not identified in *C*. *porosus*. Similarly, only a single point of insertion was identified for *pubo-ischio-femoralis internus 2* in *C*. *porosus* whereas two insertions were described for the *Caiman* [26]. The origin of the *gastrocnemius externus* in *C*. *porosus* originates from the tendon of the *caudofemoralis longus*, however in the *Alligator* [14] and the unknown crocodile species [27] it comes from the lateral condyle of the femur. Finally, the origin of e*xtensor digitorum brevis* is at the astragalus in *Alligator* [14], whereas it originates more proximally in *C*. *porosus*, on the distal fibula.

As mentioned above, only a single point of difference was found in forelimb musculature. Specifically, two tendons of origin for *triceps longus medialis* have been noted for *A*. *mississippiensis* [11] and other crocodilian taxa [20], with one tendon originating on the scapula and one from the coracoid. Only the scapular tendon was noted for *C*. *porosus*.

Digital dissection is a valuable resource for anatomical research. It is largely non-destructive and the resulting three-dimensional interactive models allow users to develop a better understanding of the position, interaction and architecture of limb muscles. It also reduces the need for traditional dissections. Presenting this information in a 3D PDF format ensures that the information is easily interpretable and accessible. Even though manually extracting the muscles from MRI scans can be time-consuming, it has significant benefits, contributing to a better understanding of anatomy [34,39]. The combination of CT and MRI data used in the present study enabled better visualisation of muscle structures and attachment sites than would the use of either in isolation. This combined method is not as common as digital dissections using contrast-enhancing agents like iodine during the CT scanning process. Contrast-enhancing methods have been used with great success in smaller specimens [32,34,35,41,44] as the resulting contrast images improves resolution and also allows for the documentation of muscle fibres [42,43]. Using MRI scans allowed us to distinguish most muscle features, however this method does limit the capacity to identify small structures like tendons. This was particularly evident when assessing the muscles of the hand and foot and in identifying attachments sites of muscles.

Digital dissection and the models resulting from this method provide a ‘visual atlas’ for interested researchers [32]. It is hoped that this ‘atlas’ will provide much needed data on the Australian estuarine crocodile, rarely considered in previous comparative studies, presumably because of difficulty in accessing specimens [22]. Presentation of a complete anatomical description and visual guide (in the form of 3D PDFs) of the appendicular myology of *Crocodylus porosus* provides essential information for anatomical and functional morphology studies focused on understanding the evolution of various locomotor strategies displayed within the Crocodylia, and archosaurs more broadly.

## Supporting information

S1 Fig Three-dimensional interactive PDF of forelimb anatomy in *Crocodylus porosus*. (PDF)

S2 Fig Three-dimensional interactive PDF of hindlimb anatomy in *Crocodylus porosus*. (PDF)
