# Supplementary figures and images for: Digital dissection and three-dimensional interactive models of limb musculature in the Australian estuarine crocodile (Crocodylus porosus)
Source: PLoS One. 2017 Apr 6;12(4):e0175079. doi: 10.1371/journal.pone.0175079 (PMC5383063; doi:10.1371/journal.pone.0175079)

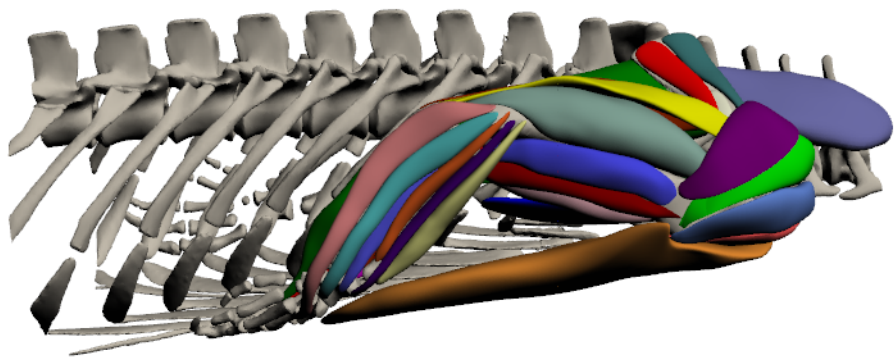

Supplement: S1 Fig — (PDF) [file pone.0175079.s001.pdf]

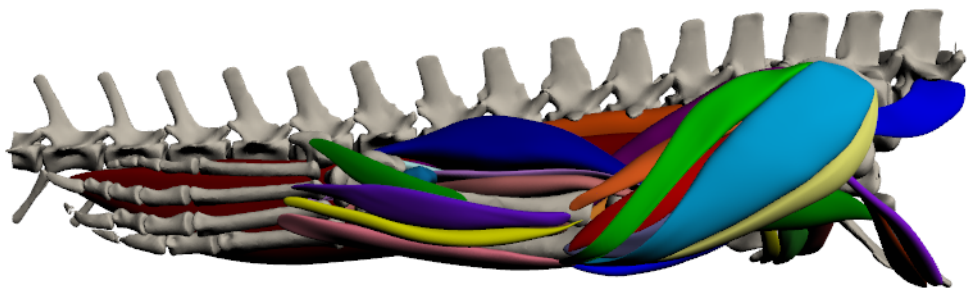

Supplement: S2 Fig — (PDF) [file pone.0175079.s002.pdf]
